# Fibrositis

**Published:** 1913-02-22

**Authors:** 


### FIBROSITIS.

At the meeting of the Balneological Section, Royal Society of Medicine, on Thursday, January 30, Dr. Llewellyn J. Lewellyn opened a discussion on fibrositis. He said he would confine his attention to so-called muscular rheumatism, or, as he preferred to term it, muscular fibrositis. "Myalgia," he thought, should be abandoned, as it predicated the absence of an anatomical lesion. "Myositis" was also misleading, because the parenchyma of the muscle was not involved, except secondarily. He inclined to the term "myofibrositis." He laid stress on the frequency with which its subjects were victims of ancestral or acquired gout, which he regarded as the chief ætiological factor. Among 1,250 cases 28 per cent. showed definite stigmata, in the shape of tophi or a classical arthritic outbreak in the great toe. An auto-toxæmia was generally responsible for the condition. Of exciting causes the chief were atmospheric changes, gastro-intestinal derangements, and prolonged muscular exertion. Neither exertion nor rest appeared to benefit the condition, but moderate exercise seemed to do so. Of 1,000 male examples, 600 were miners, labourers, gardeners, or coachmen. In its classical form the disease was a very sudden onset, usually after some slight muscular effort, but it was probably only signalising the maturing of a slow process. There was often a history of lack of the usual suppleness some days before the seizure. A rise of temperature was more often an accompaniment than was generally supposed. The salient subjective symptom was pain, often of excruciating intensity, following usually only volitional contraction. The muscle attacked by fibrositis usually increased in volume and in warmth, and there was an alteration in muscle tone. "Rheumatic" muscles were notoriously irritable, hence they readily passed into a state of spasmodic contraction, and were maintained in a state of excessive contractility. During the course of the disease the objective changes outlasted the subjective symptoms, pain being the first to disappear, then the heat and swelling, the stiffness usually remaining. The chronic form was attended by a more or less persistent aching and stiffness, with occasional exacerbations. In this form nodules developed in the muscle, or, more commonly, in the neighbourhood of its tendinous attachments. These latter were important, because of the generous supply of peripheral sense organs to the part, which made the patient aware of every slight difference of tension. These nodules served to perpetuate the muscle irritability. The pathological result was sclerosis, or an overgrowth of scar-like fibrous tissue.

* Royal Society of Medicine.

